# Characteristics of YouTube Videos About Peripheral Artery Disease During COVID-19 Pandemic

**DOI:** 10.7759/cureus.16203

**Published:** 2021-07-06

**Authors:** Corc Baytaroglu, Emrah Sevgili

**Affiliations:** 1 Cardiology, Avcilar Hospital, Istanbul, TUR

**Keywords:** covid-19, discern score, mici, peripheral artery disease, youtube

## Abstract

Introduction: To examine English language YouTube videos that covered both COVID-19 and peripheral artery disease (PAD).

Methods: The research was planned from October 1 to 5, 2020. Two cardiologists (CB and ES) executed online searches in which the term COVID-19/coronavirus was paired with common keywords about PAD, including ‘peripheral artery disease + COVID-19,’ ‘leg pain + coronavirus,’ ‘leg vascular disease + COVID-19,’ ‘atherosclerosis + COVID-19,’ and ‘claudication + coronavirus.’ For each video, a record was made of the number of days on YouTube, length, number of views and comments, and the number of ‘likes’ and ‘dislikes’. Videos were also categorized according to content as informative videos (with accurate content about the frequency of disease, symptoms, transmission, prevention techniques, and proven treatment methods), patient experience videos (with patient testimonies), or news update videos (i.e., those uploaded by professional news channels). Moreover, DISCERN and Medical Information and Content Index (MICI) were evaluated.

Results: Totally, 91 YouTube videos met study inclusion criteria. News update videos were the most-watched when compared with informative and patient experience videos (63,910 views vs 43,725 views vs19,778 views, p=0.032). The DISCERN score was significantly higher in the informative group: 2.8 for informative videos, 1.7 for patients' experience videos, and 1.8 for news update videos (p= 0.001). The most common theme was clinical symptoms in the informative videos (82.4%). The mean MICI score was calculated as 3.7±1.4 points for informative videos.

Conclusion: YouTube videos about COVID-19 and PAD are widely-viewed information sources for patients. Our study has demonstrated that YouTube videos about COVID-19 and PAD generally had poor quality content.

## Introduction

Coronaviruses are a group of enveloped RNA viruses that primarily cause respiratory system disease, including the common cold, bronchitis, pneumonia, and severe acute respiratory syndrome in mammals [1]. The new and lethal coronavirus infection, COVID-19, which originated in China, spread all around the world, infecting almost 180 million people from the beginning of the pandemic. Moreover*, *almost 3.9 million deaths due to COVID-19 and related complications have been recorded [2]. As a result, the World Health Organization declared COVID-19 a pandemic, professional healthcare workers have applied maximum effort to fighting the virus, and many hospitals were dedicated as pandemic hospitals.

Due to the ease of transmission of COVID-19, people all over the world saw public transportation limited, quarantine rules applied, and outpatient polyclinic appointments postponed. As a result, patients and their families increasingly turned to social media for medical information [3]. In this regard, Freeman and Chapman reported that social media platforms with video content were accessed significantly more often than sources with just audio and/or text content [4]. YouTube is a major social media application with billions of videos, including ones where users can find content covering medical topics like the diagnosis and treatment of various diseases. Kumar et al. investigated the contents of YouTube videos about hypertension and showed that the quality of these was insufficient [5]. Similarly, Bora et al. stated that YouTube videos about the Zika virus pandemic had inadequate content [6].

YouTube has no process for evaluating video quality, resulting in a wide variation of accuracy and utility. In the present study, our purpose was to examine English language YouTube videos that covered both COVID-19 and peripheral artery disease (PAD).

## Materials and methods

The research was planned from October 1 to 5, 2020. Approval from the Institutional Ethics Committee was not necessary because no patient data were used. Two cardiologists (CB and ES) executed online searches in which the term COVID-19/coronavirus was paired with common keywords about PAD, including ‘peripheral artery disease + COVID-19,’ ‘leg pain + coronavirus,’ ‘leg vascular disease + COVID-19,’ ‘atherosclerosis + COVID-19,’ and ‘claudication + coronavirus.’

YouTube statics show that the duration of an uploaded video is a critical factor in the video’s popularity; and previous reports claim that, in terms of popularity, the optimum length of a video is between 2 and 15 minutes [7]. Thus, only videos with a duration of between 2 and 15 minutes were included in the study. Excluded from the study were videos in languages other than English, videos unrelated to the study's subject, and videos with personal propaganda, which were classified as misleading videos. In total, 138 videos were analyzed, of which 47 were excluded from the study, leaving a YouTube playlist of 91 videos to be evaluated by the two independent cardiologists.

For each video, a record was made of its number of days on YouTube, length, number of views, comments, and number of likes and dislikes. All videos were classified as either originating from healthcare providers, nonprofessional individuals, or news agencies. Additionally, videos were classified according to whether the target audience was healthcare workers or patients. Videos were also categorized according to content as informative videos (with accurate content about frequency of disease, symptoms, transmission, prevention techniques, and proven treatment methods), patient experience videos (with patient testimonies), or news update videos (i.e., those uploaded by professional news channels).

To analyze the contents of the videos, two cardiologists completed DISCERN and Medical Information and Content Index (MICI) questionnaires. The DISCERN score provides an objective evaluation about video content quality and was calculated using a questionnaire with five yes/no questions. ‘Yes’ reflected a positive aspect and scored one point, while ‘No’ scored zero; thus, DISCERN scored videos in a range from zero to five [8]. MICI was used to assess video content in five categories: disease epidemiology, transmission, clinical symptoms, screening tests, and treatment outcomes. The MICI score for each video ranged from one to five points [9].

For the statistical analysis, the Statistical Package for the Social Sciences version 20 (SPSS IBM Corp., Armonk, NY, USA) program was used. An independent samples t-test was used to compare independent groups and χ2 and Fisher exact tests were used to compare categorical data. A post hoc test was used to compute pairwise comparisons. Inter-rater compatibility was determined by Cohen kappa score. Quantitative data were represented as mean ± standard deviation. Categorical data were noted as frequency (n) and percentages (%). The data were analyzed at a 95% confidence level and considered statistically significant if the P-value was less than 0.05.

## Results

**Figure 1:**
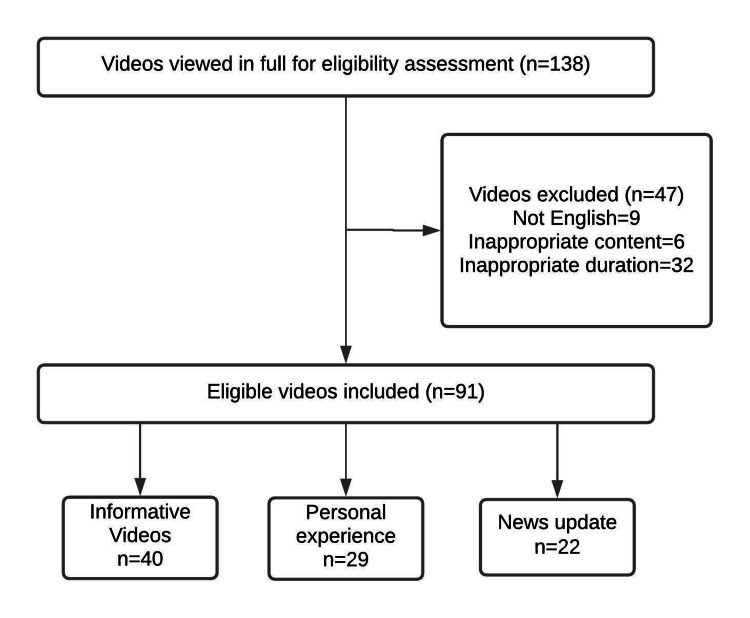
Flowchart of the study

In total, 91 YouTube videos met our inclusion criteria, while 47 videos were excluded due to inappropriate duration (32), presentation in a language other than English (9), and irrelevant content (6). Regarding the information source, 40 videos were categorized as informative, 29 videos as patient experiences, and 22 videos as news updates. The study's flow chart is found in Figure 1.

News update videos were the most-watched when compared with informative and patient experience videos (63,910 views vs 43,725 views vs 19,778 views, p=0.032). Similarly, the mean duration of videos was significantly longer in the news update group (p=0.040). In contrast, video length, views per day, likes, dislikes, and comments for each video were comparable between groups (p=0.999, p=0.717, p=0.561, p=0.419 and p=0.521, respectively). The main source of informative videos was professional healthcare providers (47.5%). All three of the video groups overwhelmingly targeted patients: 90.0% of informative videos, 86.2% of patient experience videos, and 90.9% of news update videos (p=0.838). The DISCERN score was significantly higher in the informative group: 2.8 for informative videos, 1.7 for patient experience videos, and 1.8 for news update videos (p= 0.001; Table 1).

**Table 1:** Analyses of video characteristics by usefulness category *Mean ± standard deviation.

Characteristics	Informative videos	Patient experience videos	News update videos	p-Value
Number of videos	40	29	22	
Audience interaction parameters*				
Number of views	43725.4±105464.2	19778.4±79576.4	63910.9±100722.1	0.032
Video length (min)	5.8±3.8	5.8±3.9	5.8±3.2	0.999
Duration on YouTube (days)	264.7±474.1	105.2±65.8	380.7±461.5	0.040
Views per day	288.3±1012.1	151.7±532.9	316.6±676.1	0.717
Likes	1568.3±6213.9	436.1±1124.9	934.12396.7	0.561
Dislikes	107.9±424.9	17.8±44.7	46.8±95.9	0.419
Comments	147.7±331.7	81.3±194.1	88.6±183.9	0.521
DISCERN score*	2.8±1.1	1.7±1.0	1.8±0.9	0.001
Source of upload				0.422
Professional individuals	19 (47.5%)	10 (34.5%)	5 (22.8%)	
Non-professional individuals	12 (30.0%)	11 (37.9%)	10 (45.4%)	
News agencies	9 (22.5%)	8 (27.6%)	7 (31.9%)	
Target audience				0.838
For doctors and healthcare providers	4 (10.0%)	4 (13.8%)	2 (9.1%)	
For patients	36 (90.0%)	25 (86.2%)	20 (90.9%)	

When groups are compared in regard to statically significant values, informative videos had a statically better DISCERN score when compared to patient experience and news update groups (p=0.001 and p=0.004, respectively). Additionally, the number of news update video views was significantly higher when compared to other groups (news update videos vs informative videos, p= 0.049 and news update videos vs patients experience videos, p=0.044). The duration was significantly longer for the news update group when compared to the patient experience group (p=0.033), but not significantly different when compared to the informative video group (p=0.730; Table 2).

**Table 2:** Pairwise comparisons of video groups according to usefulness Values of p<0.05 were accepted as significant and marked bold.

Characteristics	p-Value		
	Informative vs patient experience	Informative vs news update	Patient experience vs news update
Views	0.966	0.049	0.044
Duration on Youtube	0.120	0.730	0.033
DISCERN score	0.001	0.004	0.689

The most common theme in the informative videos was clinical symptoms (82.4%). A total of 43 videos (47.2%) and 45 videos (49.4%) included knowledge about disease prevalence and disease transmission, respectively. Additionally, the issues of screening tests and treatment of disease were mentioned in 53 (58.2%) and 54 videos (59.3%), respectively. The mean MICI score was calculated as 3.7±1.4 points for informative videos (Table 3). The kappa coefficient of agreement for the DISCERN score and MICI score were found as 0.87 (p<0.001) and 0.86 (p<0.001), respectively.

**Table 3:** Detailed content analysis of informative videos based on MICI scores *Mean ± standard deviation. MICI: Medical Information and Content Index.

Component of MICI scale	No. of videos with information	MICI score*
Prevalence	43 (47.2%)	0.6±0.5
Transmission	45 (49.4%)	0.6±0.5
Clinical symptoms	75 (82.4%)	0.9±0.5
Screening/tests	53 (58.2%)	0.7±0.5
Treatment/outcomes	54 (59.3%)	0.7±0.5
Total MICI score		3.7±1.4

## Discussion

In recent decades, the means of communication and accessing information have changed very rapidly. Currently, around 4.57 billion people (60% of the global population) are active internet users, and reports revealed that 95% of them watch YouTube videos [10]. At a time when it has become more challenging to access professional healthcare systems, the difficult conditions of the COVID-19 pandemic period provided an opportunity to analyze the quality of YouTube videos’ content about COVID-19 and PAD. We found that YouTube videos about COVID-19 and PAD are widely-viewed information sources but generally videos about COVID-19 and PAD have poor quality content.

The DISCERN score was developed to measure the quality of written health information and has been validated in previous studies. Ferhatoglu et al. used DISCERN scores to analyze YouTube videos about obesity surgery, concluding that videos produced by professional healthcare providers had significantly better DISCERN scores than those produced by non-professionals [11]. In another study, Yuksel and Cakmaklı used DISCERN scores to evaluate YouTube videos about COVID-19 and pregnancy, finding significantly higher scores for videos made by professional healthcare workers compared to those made by patients or news agencies [12]. Similarly, we obtained statically better DISCERN scores for informative videos compared to those of patients and new agencies (p=0.001 and p=0.004, respectively).

Nagpal et al. used MICI scores to analyze YouTube videos during the Ebola pandemic [13]. Dutta et al. investigated the role of YouTube as an information source at the global level during the COVID-19 pandemic. They analyzed the quality of YouTube videos in six languages: Arabic, Bengali, Dutch, English, Hindi, and Nigerian, finding a MICI score of 5.68 [14]. In another study, Atac et al. examined the quality of YouTube videos about COVID-19 and found a MICI score of 2.76 for English language videos and 3.33 for Turkish language videos [15]. A wide range of MICI scores are reported in the literature, perhaps because of the constant flow of new information about COVID-19, the different languages of the videos reviewed, and the different target audiences of the videos. The present research is the first to investigate the quality of YouTube videos specifically about COVID-19 and PAD, finding a MICI score of 3.7.

The number of views for YouTube videos indicates the approximate number of people reached. In the study by Yuksel and Cakmak on COVID-19 and pregnancy, news update videos had significantly more views per day in comparison to patient experience videos, but not compared to informative videos [12]. The present study found similar outcomes in regard to the number of views of YouTube videos: a significantly higher view number for news update videos about COVID-19 and PAD. The reason for this significance may have been the significantly longer duration of news agencies’ YouTube videos compared to patient experience videos.

There are some limitations of this study that is the first to analyze YouTube videos about COVID-19 and PAD. First, although we evaluated YouTube videos for the whole six-month period beginning with the first COVID-19 cases in early March, it is difficult to take into account the ongoing rapid changes in knowledge. Second, we analyzed YouTube videos in English only, and we acknowledge that video-sharing around the world is occurring in many different languages. Cross-language comparisons of the quality of YouTube videos about COVID-19 and PAD may be the subject of future study. Finally, we identified the five most common keywords to search for videos about COVID-19 and PAD; however, the inclusion of more terms related to COVID-19 and PAD may increase the number of videos.

## Conclusions

In conclusion, YouTube videos about COVID-19 and PAD are widely-viewed information sources for patients. Our study has demonstrated that YouTube videos about COVID-19 and PAD generally have poor quality content but, with improvement, YouTube videos could potentially become effective and accessible sources of information about COVID-19 and PAD.
